# Properties of *Halococcus salifodinae*, an Isolate from Permian Rock Salt Deposits, Compared with Halococci from Surface Waters

**DOI:** 10.3390/life3010244

**Published:** 2013-02-28

**Authors:** Andrea Legat, Ewald B. M. Denner, Marion Dornmayr-Pfaffenhuemer, Peter Pfeiffer, Burkhard Knopf, Harald Claus, Claudia Gruber, Helmut König, Gerhard Wanner, Helga Stan-Lotter

**Affiliations:** 1 Department of Molecular Biology, University of Salzburg, Billrothstr. 11, 5020 Salzburg, Austria; E-Mails: Andrea.Legat@gmx.at (A.L.); marion.dornmayr@gmail.com (M.D.-P.); Claudia.Gruber@sbg.ac.at (C.G.); 2 Medical University Vienna, Währingerstrasse 10, 1090 Wien, Austria; E-Mail: dennerebm@yahoo.de; 3 Institute of Microbiology and Wine Research, Johannes Gutenberg-University, 55099 Mainz, Germany; E-Mails: ppfeiffe@uni-mainz.de (P.P.); hclaus@uni-mainz.de (H.C.); hkoenig@uni-mainz.de (H.K.); 4 Frauenhofer-Institut für Molekularbiologie und Angewandte Ökologie, 57392 Schmallenberg, Germany; E-Mail: burkhard_knopf@hotmail.com; 5 LMU Biocenter, Ultrastructural Research, Grosshadernerstrasse 2-4, 82152 Planegg-Martinsried, Germany; E-Mail: Wanner@lrz.uni-muenchen.de

**Keywords:** *Halococcus *species, *Halococcus salifodinae*, haloarchaea, Permian salt deposit, cell wall polymer, polyhydroxyalkanoate, prokaryotic evolution

## Abstract

*Halococcus salifodinae* BIp^T^ DSM 8989^T^, an extremely halophilic archaeal isolate from an Austrian salt deposit (Bad Ischl), whose origin was dated to the Permian period, was described in 1994. Subsequently, several strains of the species have been isolated, some from similar but geographically separated salt deposits. *Hcc. salifodinae *may be regarded as one of the most ancient culturable species which existed already about 250 million years ago. Since its habitat probably did not change during this long period, its properties were presumably not subjected to the needs of mutational adaptation. *Hcc. salifodinae* and other isolates from ancient deposits would be suitable candidates for testing hypotheses on prokaryotic evolution, such as the molecular clock concept, or the net-like history of genome evolution. A comparison of available taxonomic characteristics from strains of *Hcc. salifodinae* and other *Halococcus* species, most of them originating from surface waters, is presented. The cell wall polymer of *Hcc. salifodinae* was examined and found to be a heteropolysaccharide, similar to that of *Hcc. morrhuae*. Polyhydroxyalkanoate granules were present in *Hcc. salifodinae*, suggesting a possible lateral gene transfer before Permian times.

## 1. Introduction

*Halococcus salifodinae* BIp^T^ DSM 8989^T^ was obtained as a viable isolate from Permian rock salt deposits of a mine in Bad Ischl, Austria [1,2]. The strain grew optimally at a salinity of 20%–25%, a pH value of 7.4 and at 40 °C. Subsequently, several halococcal strains were isolated from similar sites in England and Germany, which had identical 16S rRNA gene sequences and numerous similar properties as the Bad Ischl strain BIp^T^ [3].

The genus *Halococcus *[4], emended by Oren *et al.* [5] currently comprises seven formally described species, which are listed here with their sites of isolation and reference in brackets: *Hcc. morrhuae *(seawater, saline lakes, salterns and salted products, [6]), *Hcc. saccharolyticus *(marine salterns, [7]), *Hcc. salifodinae *(rock salt from mines in Germany and Austria, also from brine in a salt mine in England, [1]), *Hcc. dombrowskii *(bore core from a salt mine in Austria, [8]), *Hcc. hamelinensis *(stromatolites of Shark Bay, Hamelin Pool in Western Australia, [9]), *Hcc. qingdaonensis* (crude sea-salt sample collected near Qingdao in Eastern China, [10]) and *Hcc. thailandensis* (fermented fish sauce produced in Thailand) [11]. Thus, two species—*Hcc. salifodinae* and *Hcc. dombrowskii* - were isolated from Permo-Triassic salt sediments, whereas the other five species can be regarded as inhabitants of hypersaline surface waters or heavily salted products. 

**Figure 1:**
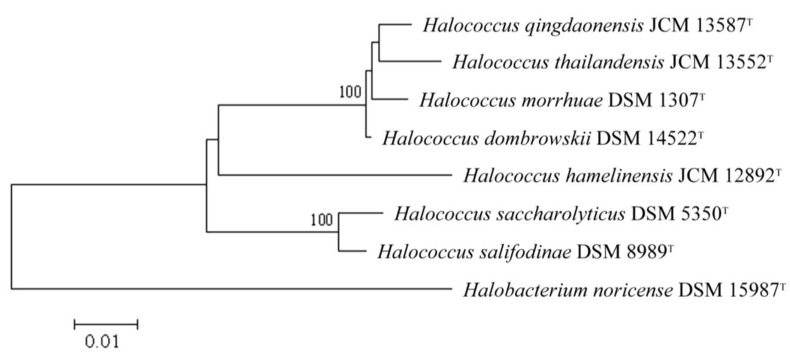
Distance-matrix neighbor-joining tree, showing the phylogenetic relationships of *Halococcus *type strains. The tree is based on an alignment of 16S rRNA gene sequences. Bootstrap values higher than 70 out of 1000 subreplicates are indicated at the respective bifurcations. The tree was constructed using the neighbour-joining method of Saitou and Nei [13]. The bar represents the scale of estimated evolutionary distance (1 % substitutions at any nucleotide) from the point of divergence. *Halobacterium noricense *was used as an outgroup.

A study by Wright [12] using 16S rRNA gene sequences of 61 haloarchaeal taxa, revealed that the mean genetic divergence over all possible pairs of halophilic archaeal 16S rRNA gene sequences was 12.4 ± 0.38%, indicating close relatedness. In comparison, the greatest genetic divergence within methanogenic archaea was 34.2% [12]. Within the halophilic archaea, *Halococcus *species form an even closer related group (see Figure 1), with 16S rRNA gene sequence similarities of 98.2%–98.7% between *Hcc. thailandensis*, *Hcc. morrhuae*, *Hcc. qingdaonensis* and *Hcc. dombrowskii*, and somewhat lower similarities of 93.7%–94.1% between *Hcc. hamelinensis*, *Hcc. saccharolyticus* and *Hcc. salifodinae* [11].

Our notions on prokaryotic evolution and evolution in general have been shaped by the concept of a molecular clock, which suggests an approximately uniform rate of molecular evolution among species and duplicated proteins over time [14]. Although subject to various criticisms, molecular-clock techniques still remain the only way to infer the timing of gene duplications and speciation events in the absence of fossil or biogeographical records [14]. The concept was applied previously to date the sequence divergences of halophilic archaeal protein-encoding genes, compared to the divergence of homologous non-halophilic eubacterial protein-encoding genes, assuming a point of haloarchaeal species diversion of 600 million years before present [15]. However, modern results from genome sequences revealed a much more complex history of life than can be depicted in bifurcating trees [16]. Widespread horizontal gene transfer—although occurring to different extents—, endosymbioses, gene losses and other processes cause the presence of different molecules with different histories in a species, and members of the same species were found to differ dramatically in gene content, leading to the suggestion of a fuzzy species concept in prokaryotes [16].

Some of these problems and uncertainties might be resolvable when viable microorganisms from well-dated ancient geological sites would be compared on a molecular basis with contemporary species. A crucial issue is the proof that microorganisms from ancient materials, like million year old deep subseafloor sediments, or Permian salt evaporites, are as old as the geological sites from which they were obtained (see [17,18,19] for discussions). The determination of the age of a single average bacterium is not possible with currently available methods, since its mass is only about a picogram. Thus, claims of ancient microorganisms were often dismissed as being due to laboratory contaminations. 

Recently, small particles of about 0.4 μm in diameter were imaged by microscopy directly within fluid inclusions of 22,000–34,000 year old salt bore cores and, following successful culturing, identified as haloarchaea [18,20]. Embedding of halophilic microorganisms in fluid inclusions upon formation of salt crystals is well known, and fluid inclusions have been suggested as sites for preservation of microbial life [21,22,23]. In addition, Gramain *et al.* [24] reported isolation of haloarchaea from well-dated salt bore cores of Pliocene age (5.3 to 1.8 million years). Thus there is a growing body of evidence that haloarchaea survive for great lengths of time [24].

Here we review the properties of coccoid haloarchaea isolated from Permo-Triassic salt sediments, and relate them to those of halococci, which were isolated from surface waters. In addition, new data on *Halococcus salifodinae* concerning the chemical composition of its cell wall are included as well as DNA-DNA hybridization experiments between several strains of the species. Recently, the first genome sequence of a halococcus, *Hcc. hamelinensis* 100A6^T^, became available [25] and therefore information for several genes (*phaC* synthases; subunit A of the rotary A-ATPase) is examined here for their potential use in delineating the evolution of haloarchaeal cocci.

## 2. Results and Discussion

### 2.1. General Description of halococci [26]

**Figure 2:**
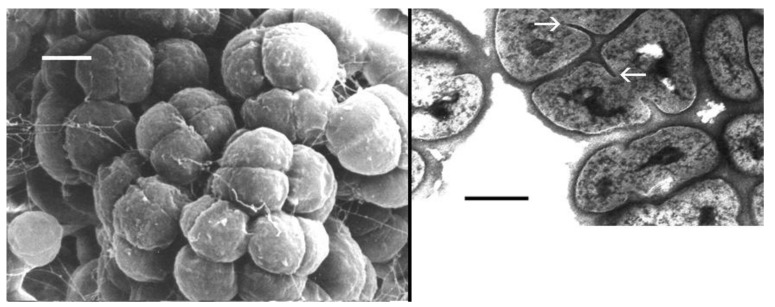
Left panel: Scanning electron micrograph of *Halococcus salifodinae* Br3 (DSM 13046), grown in liquid culture medium [1]. Bar, 500 nm. Right panel: Transmission electron micrograph of an ultrathin section of *Halococcus salifodinae* BIp^T^ DSM 8989^T^. Cells are surrounded by an amorphous layer of wall material. Septum formation is visible (white arrows). Bar, 760 nm.

Halococci are cells of 0.8-1.5 μm diameter, occurring in pairs, tetrads, sarcina packets, or large clusters [1,26]; see Figure 2, left panel. A striking difference to other genera of the *Halobacteriaceae* is their resistance to lysis in water (or generally hypotonic solutions). They are non-motile, strictly aerobic and extremely halophilic, requiring at least 2.5 M NaCl for growth and 3.5–4.5 M NaCl for optimum growth [26]. Their optimum growth temperature is between 30-40 °C but most strains can grow up to 50 °C. 

### 2.2. Properties of Isolates from Permo-Triassic Salt Sediments and Surface Waters

Following the formal description of *Halococcus salifodinae* BIp^T^ DSM 8989^T^ as a novel species from a Permian salt deposit [1], a detailed comparison with similar isolates from a British halite formation (strain Br3) and from a bore core of the salt mine in Berchtesgaden, Germany (strain BG2/2) was undertaken [3]. In addition, two further isolates (strains H2, N1) from the Bad Ischl salt mine were similar enough to *Hcc*. *salifodinae *BIp^T ^to consider them strains of the same species, obtained 8 years after the initial rock salt samples were taken [3]. The sequences of the 16S rRNA genes of all five strains were identical, as were their polar lipid composition, colonial and cellular morphology, cell size, cellular arrangement, and pigmentation. Strong similarities were found between whole-cell protein patterns, G+C contents, growth characteristics, enzyme content and susceptibility to antibiotics. Table 1 provides a comparison of biochemical characteristics of the five strains of *Hcc. salifodinae* (numbered 1–5) with the other presently known six halococcal species (numbers 6–11). All strains of *Hcc. salifodinae *were positive for alkaline phosphatase, esterase (C4), esterase lipase (C8), oxidase and catalase (Table 1). Variable reactions among strains were observed for acid phosphatase, N-acetyl-β-glucosaminidase, nitrate reduction, hydrolysis of Tween 20 and gelatine liquefaction (Table 1). Starch and Tween 80 were hydrolysed by strains BIp^T^, BG2/2 and Br3, but casein was not. Goh * et al.* [9] reported that the two isolates of *Hcc. hamelinensis* were negative for oxidase activity, whereas *Hcc. morrhuae* NRC 16008, *Hcc. saccharolyticus* ATCC 49257^T^ and *Hcc salifodinae* DSM 8989^T^ were all positive. The API ZYM strips revealed that the two isolates of *Hcc. hamelinensis* were positive for leucine arylamidase, but negative for trypsin, as were all other halococci.

**Table 1:** Characteristics of five independently isolated strains of *Halococcus salifodinae *from three different locations [3] and other *Halococcus* species. 1, *Hcc. salifodinae* BIp^T ^DSM 8989^T^, type strain; 2, *Hcc. salifodinae *BG2/2 (DSM 13045); 3, *Hcc. salifodinae *Br3 (DSM 13046); 4, *Hcc. salifodinae *H2 (DSM 13071); 5, *Hcc. salifodinae* N1 (DSM 13070); 6, *Hcc. saccharolyticus* DSM 5350^T^ (data from [26]); 7, *Hcc. hamelinensis *JCM 12892^T^ (data from [9]*)*; 8, *Hcc. thailandensis* (data from [11]); 9, *Hcc. dombrowskii DSM *14522^T^ (data from [8]); *10, Hcc. qingdaonensis *JCM 13587^T^ (data from [10]); *11, Hcc. morrhuae *DSM 1307^T^ (data from [8,26]).

Characteristic*	1	2	3	4	5	6	7	8	9	10	11
Oxidase	+	+	+	+	+	+	-	+	+	-	+
Catalase	+	+	+	+	+	+	+	+	+	+	+
Alkaline phosphatase	(+)	+	+	+	+						
Esterase (C4)	+	+	+	+	+						
Lipase esterase (C8)	+	+	+	+	+						
Lipase (C14)	-	-	-	-	-						
Leucine arylamidase	-	-	-	-	-		+				
Trypsin	-	-	-	-	-		-				
Acid phosphatase	-	-	+	+	+				+		-
Cystine arylamidase	-	-	-	-	-				+		-
Nitrate reduction	+	+	+	-	+	+		+	+		+
Gelatin liquefaction	+	-	-			v	-	-	+	-	v
Hydrolysis of starch	+	+	+			-	+	-			v
casein	-	-	-			-		-			
Tween 20	-	+	+					-			
Tween 80	+	+	+			-				-	+
Sensitivity to anti-biotics: Tetracycline	+	+	+	+	+	-	-	-	-	-	-
" : Chloramphenicol	+	+	+	+	+	-		-	-	+	-
" : Novobiocin	+	+	+	+	+	-	+	+	+		+

* +, positive reaction; -, negative reaction; (+) weak reaction; v, variable; empty box: no data available.

The results confirmed the assignment of strains 1–5 from salt deposits to the same species, *Hcc*. *salifodinae*. *Hcc*. *salifodinae *is distinct from other halococci, but, based on 16S rRNA sequences, appeared phylogenetically more closely related to *Hcc*. *saccharolyticus*. The similarity of the 16S rDNA sequence of *Hcc. salifodinae *BIp^T^ DSM 8989^T ^to that of *Hcc. saccharolyticus *DSM 5350^T^ was 98.9%. A similarity value of >97% necessitates the determination of the DNA-DNA homology by hybridization experiments, in order to delineate the identity of species [27]. Therefore, DNA-DNA hybridization between *Hcc. salifodinae* BIp^T^ DSM 8989^T^ and *Hcc. saccharolyticus *DSM 5350^T ^was performed and showed a value of 63.6%. DNA-DNA hybridization data confirmed that the two strains represent two different *Halococcus *species*,* since it is accepted that strains of a single species exhibit ≥ 70% DNA relatedness [28]. DNA-DNA hybridization was also carried out among the five *Hcc. salifodinae* strains and revealed values in the range of 82.6% to 95.0%, corroborating the assignment of the strains to a single species. Thus it was demonstrated that in geographically separated halite deposits—located in Austria, Germany and England—of similar geological age, identical species of halococci are present. It can therefore be speculated that their native environment may have been the ancient Zechstein sea, spreading over large parts of what is now Europe [29] and it is tempting to suggest that they might be marker organism for salt deposits from certain geological periods [30].

### 2.3. Cell Wall of Hcc. salifodinae

The cell wall of *Halococcus* species is very prominent as seen in TEM micrographs (Figure 2, right panel; [1,3]) with a thickness of 50–60 nm reported for *Hcc. morrhuae* [31]. The material appears amorphous and the formation of septa is visible (white arrows). 

So far extremely halophilic Archaea are known to have developed three different cell wall types: (a) S-layers [32], (b) a heteropolysaccharide [33] and (c) a glutaminylglycan [34]. Layers of (glyco-) protein subunits (S-layers) represent the most common cell wall structures among Archaea [35,36]. The occurrence of S-layers, which were found in extremely halophilic archaeal genera such as *Halobacterium *and *Haloferax *[32] or several methanogenic genera such as *Methanococcus *[37] and *Methanothermus *[38] in the Euryachaeota branch, was not detected in *Halococcus salifodinae*. The chemical composition of only two cell walls from coccoid haloarchaea has been reported to date:* Hcc. morrhuae* CCM 859 possesses a heteropolysaccharide [33] and the haloalkalophile *Natronococcus occultus* contains a glutaminylglycan [34]. No molecular structures of these heteropolysaccharides are available yet. Since *Hcc. salifodinae* BIp^T^ originated from an ancient habitat existing for about 250 million years, the cell surface structure of the organism was of special interest and the chemical composition of its cell wall was analyzed. 

No protein, as in S-layers, was found but instead, different monosaccharides were identified as constituents of the cell wall polymer, as was the case with the closely related species *Halococcus morrhuae *[33,39,40,41]. The results of the chemical analysis are summarized in Table 2. These data showed that the cell wall composition of *Halococcus salifodinae *is very similar to that of *Halococcus morrhuae*. Both cell wall polymers are composed of the neutral sugars galactose, mannose and glucose, the amino sugars glucosamine and galactosamine and the uronic acids glucuronic acid and galacturonic acid. Glycine and lysine were the only amino acids which could be detected in small amounts. However, the molar ratios of the cell wall constituents differed significantly between *Hcc. morrhuae *und *Hcc. salifodinae*.

**Table 2:** Chemical composition of the cell wall polymer of *Halococcus morrhuae *and *Halococcus salifodinae*.

Cell wall constituents^a^	*Hcc. morrhuae* CCM 859^b^	*Hcc. salifodinae* BIp^T^ DMS 8989^T^
Glucose	440	470
Mannose	350	220
Galactose	270	360
Ribose	n.d.	60
Glucosamine	380	180
Galactosamine	200	80
Glucuronic acid	470	60
Galacturonic acid	200	20
Gulosaminuronic acid	110	n.d.
Acetate	620	660
Sulfate	1470	1580
Phosphate	120	130
Glycine	100	7
Lysine	n.d.	1

^a^ nmol/mg cell wall (dry weight); ^b^ data from [33,42]; n.d., not determined

These results allow us to speculate that a heteropolysaccharide forms the main cell wall polymer of *Halococcus salifodinae, *as was described for *Halococcus morrhuae *[33]. The carbohydrates of the cell wall sacculi of *Halococcus morrhuae *are arranged in three domains. These three domains are partly linked by N-glycyl-glucosaminyl bridges [41]. The data suggested that *Halococcus salifodinae*, a viable isolate from Permian rock salt deposits, has not developed a novel cell wall type, but possesses most likely a similarly structured, but modified heteropolysaccharide as the closely related species *Halococcus morrhuae*.

The coccus is the simplest of possible cell shapes. The coccoid morphology has been suggested as the first (bacterial) cell morphology [43], but later, arguments for a rod-shaped morphology were viewed as more likely, mainly a better surface to volume ratio, providing an increased area for uptake of nutrients [44]. Still, this issue has not been solved yet, and it will be interesting to see a comparison of the synthesis of peptidoglycan, the nearly ubiquitous cell wall polymer of bacteria, which is lacking in halococci [39], with that of heteropolysaccharides. Peptidoglycan in Gram-positive cells consists of a thick layer of several nm width, similar to the heteropolysaccharide layer of halococci [39]. Both polymers appear functionally identical, providing stability to the cell, both form septa during cell division (in contrast to constrictions of most Gram-negative bacteria), but, judging from their different compositions, their biochemical pathways must involve quite a different set of enzymes. 

### 2.4. Production of polyhydroxyalkanotes (PHA)

Due to the considerable biotechnological and medical potential, the pathways of bacterial synthesis of polyhydroxyalkanotes (PHA) have been examined intensely (for a review see [45]). Accumulation of PHAs by haloarchaea was first reported by Fernandez-Castillo *et al.* [46]). So far, the best PHA producer of the family *Halobacteriaceae* is *Haloferax mediterranei* (see [47]). Recently, evidence for the production of polyhydroxyalkanoates by halococci was published ([48]), which included staining and electron microscopy of PHA granules as well as chemical identification by NMR from *Hcc. morrhuae* DSM 1307^T^, *Hcc. saccharolyticus* DSM 5350^T^, *Hcc. salifodinae* BIp^T^ DSM 8989^T^, *Hcc. dombrowskii* DSM 14522^T^, *Hcc. hamelinensis* JCM 12892^T^ and *Hcc. qingdaonensis* JCM 13587^T^. Genetic information about haloarchaeal PHA synthases is still sparse. Of interest is the finding that high homologies exist to the bacterial enzymes as reported by several authors [47,49,50,51,52]. In a phylogenetic tree of PHA polymerases (*phaC* synthases), bacterial and haloarchaeal sequences clustered together, and the most closely affiliated microorganisms shared habitats of marine origin [47]. These observation suggested horizontal gene transfer [53]. It was even proposed that *phaC* synthases belonging to Class III of halophilic and non-halophilic microorganisms may have had a common ancestor [47].

### 2.5. BLAST Search of Genes in the Genome of Hcc. hamelinensis.

The genome sequence of *Hcc. hamelinensis* 100A6^T^ has recently been published [25]. This allows preliminary comparisons with the genome content of other halococci, since as noted above, all halococci appear to be closely related. BLAST (Basic Local Alignment Search Tool [54]) searches were carried out with two examples, *phaC* (see 2.4.) and subunit A of the haloarchaeal ATPsynthase.

#### 2.5.1. Polyhydroxyalkanaote Synthase (*phaC*)

The gene coding for *phaC* (polyhydroxyalkanaote synthase) from *Haloferax mediterranei*, accession number ACB10370 [52] was used with the program TBLASTN. The identities for the (translated) protein (492 amino acids) were 56%, similarities were 71%. A search with the nucleotide sequence for the related *phaC* (1425 nucleotides) of *Haloarcula hispanica *ATCC 33960 [52] resulted in 77% identities in the genome sequence of *Hcc. hamelinensis* 100A6^T^.

#### 2.5.2. Subunit A (*atpA*) of the Archaeal ATP Synthase

Fundamental enzyme complexes in all cells are the rotary ATP synthases/ATPases, which catalyze the synthesis of ATP at the expense of a proton or ion gradient and include three related members (for a recent review see [55]). The A-ATP synthase is present in archaea, which is similar to the eukaryotic V-ATPases. Preliminary information of the occurrence of A-ATPases in isolates from Permian salt sediments was obtained by immunological and biochemical properties with strain 54R, a close relative of the rod-shaped *Halorubrum saccharovorum *[56], whose ATPase has been characterized in detail [57,58]. However, no information exists yet about rotary ATPases from halococci. A BLAST search with the nucleotide sequence of subunit A of the A-ATPase from *Halobacterium salinarum * strain NRC-1 (NC_002607.1; length of 1758) revealed an identity of 81% in the genome sequence of *Hcc. hamelinensis* 100A6^T^.

### 2.6. Are Permo-Triassic Isolates Suitable for Evolutionary Studies?

#### 2.6.1. What Type of Results Can be Expected?

Using 16S rRNA sequences as a chronometer, Dennis and Shimmin [15] estimated that *Halobacterium*, *Haloferax*, and *Haloarcula *diverged from a common ancestor about 600 × 10^6 ^years ago. This calculation was based on the assumption of a constant and uniform rate of sequence diversions of 1% per 50 × 10^6^ years [59]. This time frame appears rather short, since evidence (although disputed) for haloarchaeal DNA was found in Silurian salt sediments (416–429 million years old), some from *Halobacterium* species and some of unknown affiliation [60], perhaps from older and now extinct microorganisms. This type of questions may be answerable, *i.e*., the molecular clock concept could perhaps be verified, at least with highly conserved genes, when genome sequences of strains of Permo-Triassic origin are available. The evolution of biochemical pathways for cell wall synthesis or production of PHAs could be clarified (see above). Also, insights into the evolution of very complex cellular systems, e.g., ATP synthases, could be gained. Other expected results should be information about gene losses (see [16]), which could be detected with sequenced genomes of "ancient" subsurface prokaryotes. This could then explain unexpected phylogenetic results, for example, distribution of genes which do not fit a tree [16]. More information about horizontal gene transfer should probably become available.

#### 2.6.2. Which Strains Should be Used for Comparative Studies?

Comparative 16S rRNA gene sequence analyses showed a similarity of 98.9% between *Hcc. salifodinae* BIp^T^ DSM 8989^T^ and *Hcc. saccharolyticus* DSM 5350^T^. Thus, *Hcc. saccharolyticus* DSM 5350^T^ would appear to be an appropriate counterpart for *Hcc. salifodinae* BIp^T^ DSM 8989^T^ to carry out comparisons between a "contemporary" and a "Permian" genome, although the strains belong to different species, due to their DNA-DNA hybridization values of < 70% and several different phenotypic properties. A description of isolates from surface waters of salterns in Goa, India, showed other suitable candidates: halococcoid isolates were found with 98–99% similarities of 16S rRNA genes to *Hcc. salifodinae* and *Hcc. saccharolyticus* [61]. From salt mines in Turkey, two halococcoid strains were obtained with 16S rRNA similarities of 99.8% and 99.3%, respectively, to *Hcc. dombrowskii*, and one pleomorphic strain with 99.7% similarity to *Hbt. noricense* [62]. Both *Hcc. dombrowskii* and *Hbt. noricense* originated also from Permo-Triassic salt sediments [8,63]. Detailed descriptions of all strains and whole genome sequences should yield meaningful comparisons. 

## 3. Experimental Section

*Growth of microorganisms*: *Halococcus salifodinae* strains Blp^T^ (DSM 8989^T^), BG2/2 (DSM 13045), Br3 (DSM 13046), N1 (DSM 13070), H2 (DSM 13071), *Hcc. morrhuae* DSM 1307^T^, *Hcc. saccharolyticus *DSM 5350^T^ and *Hcc. dombrowskii* DSM 14522^T^ were grown in modified M2 medium as described by Denner *et al.* [1], which contained (g/l): yeast extract (5.0), casamino acids (5.0), NaCl (200.0), Tris/HCl (12.1), MgCl_2_ x 6 H_2_O (20.0), CaCl_2_ x 2 H_2_O (2.0) and KCl (0.2) at pH 7.4. For testing the presence of polyhydroxyalkanoates, strains were also grown in synthetic medium with 1% (w/v) glucose [64], except that KBr was used instead of NaBr. *Hcc. hamelinensis* JCM 12892^T^ and *Hcc. qingdaonensis* JCM 13587^T^ were grown in complex medium (DSM no. 372, http://www.dsmz.de/microorganisms/medium/pdf/DSMZ_Medium372.pdf). All cultures were incubated at 37 °C with shaking at 180 rpm in an Innova 4080 incubator (New Brunswick Scientific).

*Preparation and analysis of cell walls:* For preparation of cell walls, cultures were harvested by centrifugation at 8000 rpm for 20 min. The cell pellet was washed three times with deionized water and cells were disrupted with glass beads (Ø 0.5–0.7 mm) in a Braun cell homogenisator (model MKS) for 20 min. The crude cell wall preparation was incubated overnight with trypsin (0.5 mg/ml; Merck) in a 0.05 M (pH 7.8) potassium phosphate buffer at 37 °C. Cell wall preparations were washed four times with deionized water and freeze-dried (Lyovac, Leybold-Heraeus). Neutral sugars and uronic acids were released from cell walls under vacuum with 2 M HCl at 100 °C for 2 h and 3 h, respectively; amino sugars with 4 M HCl at 100 °C for 16 h. After removal of the acid the aqueous uronic acid solution was adjusted to pH 9 with an ammonia solution (1 M) and incubated for 2 h at room tem­perature to delactonize. The compounds were identified and quantified by HPLC using a CarboPac^®^PA1 column (Dionex) and pulsed amperometric detection. Monosaccharides and amino sugars were separated with a gradient reaching from 16 to 300 mM NaOH; for separation of uronic acids a gradient consisting of (A) 100 mM NaOH and (B) a solution of 100 mM NaOH with 1 M sodium acetate was used. Acetate [65], sulfate [66] and phosphate [67] were determined by the described methods.

*Enzyme tests:* The API ZYM system (bioMerieux) was used for the identification of 19 enzymatic activities [68]. Test strips were inoculated with cells in the exponential phase of growth and were incubated at 37–39 °C for up to 24 h [8]. Standard tests (oxidase, catalase, nitrate reduction, gelatin liquefaction, hydrolysis of starch, casein and Tween) were performed as described previously [8] or as recommended by Oren *et al.* [69]. Tests were performed at least in triplicate.

*DNA-DNA hybridization*. DNA was isolated as described by Cashion *et al.* [70]. Levels of DNA-DNA hybridization were determined spectrophotometrically by the renaturation method of De Ley *et al.* [71], with the modifications by [72] and [73]. Renaturation rates were computed by the program TRANSFER.BAS [74]. These experiments were carried out by the Identification Service of the DSMZ, Braunschweig, Germany. 

*Other methods: *For comparative phylogenetic analyses 16S rRNA gene sequences from validly described *Halococcus *spp. were obtained from the European Molecular Biology Laboratory (EMBL) web interface and fitted in a subset of aligned archaeal sequences obtained from the Ribosomal Database Project II [75]. Phylogenetic relationships of the sequences were constructed by using distance-matrix methods (corrections as in Jukes and Cantor [76]). The web-based software MEGA 2 (http://www.megasoftware.net; [77]) and Clustal X [78] were used for sequence analysis and for construction of the phylogenetic tree, including maximum-likelihood and maximum parsimony methods. Confidence of the branching patterns was assessed by bootstrap analysis (1000 replicates). Scanning and transmission electron microscopy was performed as described previously [1,3]. 

## 4. Conclusions

From considerations of close genetic relatedness and origin from ancient geological materials it is concluded that *Halococcus salifodinae* strains can be considered as living fossils and constitute a promising source of novel evolutionary information.
